# A role of ghrelin in canine mammary carcinoma cells proliferation, apoptosis and migration

**DOI:** 10.1186/1746-6148-8-170

**Published:** 2012-09-23

**Authors:** Kinga Majchrzak, Karol M Pawłowski, Emilia J Orzechowska, Izabella Dolka, Joanna Mucha, Tomasz Motyl, Magdalena Król

**Affiliations:** 1 Department of Physiological Sciences, Faculty of Veterinary Medicine, Warsaw University of Life Sciences - WULS, Nowoursynowska 159, Warsaw, 02-776, Poland; 2 Department of Molecular Biology, Faculty of Biology, Warsaw University, Miecznikowa 1, Warsaw, Poland; 3 Department of Pathology and Veterinary Diagnostics, Faculty of Veterinary Medicine, Warsaw University of Life Sciences - WULS, Nowoursynowska 159, Warsaw, 02-776, Poland

**Keywords:** Canine mammary carcinoma, Ghrelin, Growth hormone secretagogue receptor

## Abstract

**Background:**

Ghrelin is a natural ligand of the growth hormone secretagogue receptor (GHS-R). They are often co-expressed in multiple human tumors and related cancer cell lines what can indicate that the ghrelin/GHS-R axis may have an important role in tumor growth and progression. However, a role of ghrelin in canine tumors remains unknown. Thus, the aim of our study was two-fold: (1) to assess expression of ghrelin and its receptor in canine mammary cancer and (2) to examine the effect of ghrelin on carcinoma cells proliferation, apoptosis, migration and invasion. The expression of ghrelin and its receptor in canine mammary cancer tissues and cell lines (isolated from primary tumors and their metastases) was examined using Real-time qPCR and immunohistochemistry. For apoptosis analysis the Annexin V and propidium iodide dual staining was applied whereas cell proliferation was evaluated by MTT assay and BrdU incorporation test. The influence of ghrelin on cancer cells migration and invasion was assessed using Boyden chamber assays and wound healing assay.

**Results:**

The highest expression of ghrelin was observed in metastatic cancers whereas the lowest expression of ghrelin receptor was detected in tumors of the 3^rd^ grade of malignancy. Higher expression of ghrelin and its receptor was detected in cancer cell lines isolated from metastases than in cell lines isolated from primary tumors. *In vitro* experiments demonstrated that exposure to low doses of ghrelin stimulates cellular proliferation, inhibits apoptosis and promotes motility and invasion of canine mammary cancer cells. Growth hormone secretagogue receptor inhibitor ([D-Lys^3^]-GHRP6) as well as RNA interference enhances early apoptosis.

**Conclusion:**

The presence of ghrelin and GHS-R in all of the examined canine mammary tumors may indicate their biological role in cancer growth and development. Our experiments conducted *in vitro* confirmed that ghrelin promotes cancer development and metastasis.

## Background

Ghrelin was identified in human and rat stomach in 1999 by Kojima et al. [1] as endogenous ligand for growth hormone secretagogue receptor type 1a (GHS-R1a). It belongs to the G protein coupled receptor family and is widely distributed along various tissues. GHS-R has two variants of splicing: functional type 1a and truncated type 1b, which arises from an alternative splicing and its function is still unclear [2,3].

The GHS-R activation in pituitary results in considerably increased secretion of growth hormone (GH). This is the main effect of ghrelin activity in humans and in animals. Ghrelin is a highly conserved peptide among various species [1,4]. It is mainly produced by endocrine X/A-like cells of stomach’s submucosal layer and, secreted directly into the blood [5]. However, the presence of ghrelin has also been detected in other areas of gastrointestinal tract, as well as in many other tissues [6].

In healthy dogs, ghrelin regulates feeding behavior and energy metabolism. Plasma ghrelin levels increase before feeding time, and decrease after eating. These changes are associated with the insulin and glucose concentration [7,8]. Some studies on dogs suggested role of ghrelin in the development of adipositas due to higher circulating levels of ghrelin in obese dog than in normal or lean animals [7,9].

The ghrelin expression (and its receptor) has also been observed in multiple endocrine and non-endocrine tumors and related cell lines in humans, such as: pituitary adenomas, thyroid follicular cancer and parathyroid adenomas, pancreatic-related endocrine tumors, oral squamous cell carcinoma, gastric carcinoids, colon cancer, renal carcinoma, bronchial carcinoid, testicular and ovarian tumors, adrenocortical tumors, prostate cancer and breast cancer [10]. The co-expression of ghrelin and its receptor in various tumors and cancer cell lines may indicate their autocrine/paracrine role in the tumor growth and development. Thus, the effect of ghrelin on cancer cells was investigated in multiple *in vitro* experiments in humans and rats, however the result are contradictory [11-22].

The role of ghrelin/GHS-R axis in tumor initiation, progression and metastasis in animals is still unclear. As far as we realize, there are no published studies about the expression and function of ghrelin in canine tumors. Our previous studies have shown that cell lines with the highest proliferative potential have high expression of GHS-R [23]. Moreover, we found its up-regulation in metastatic cancer cell lines (isolated from canine mammary cancer metastases to the lungs) what may indicate the role of ghrelin and GHS-R in metastasis [24]. This study was therefore designed to characterize the expression of ghrelin and its receptor in canine mammary cancer tissues and cancer cell lines. We have also examined the effect of ghrelin on cancer cells proliferation, apoptosis, migration and invasion.

## Methods

### Cell culture

The cell lines used for this study have previously been given an account of Król et al. [23-25]. Two adenocarcinoma cell lines isolated from the canine mammary gland tumors (CMT-W1 and CMT-W2) and two cell lines isolated from their lung metastases (CMT-W1M and CMT-W2M) were kindly donated by Prof. Dr. Maciej Ugorski and Dr. Joanna Polanska from Wroclaw University (Poland). The cell lines were cultivated in RPMI-1640 medium containing 10% heat-inactivated fetal bovine serum (FBS), 50 U/ml penicillin, 50 μg/ml streptomycin, 2,5 μg/ml amphotericin B (reagents obtained from Sigma Aldrich Chemical Co., USA) and grown in tissue culture flasks (Nunc™, Denmark) in an atmosphere of 5% CO2 and 95% humidified air at 37°C, and routinely subcultured every second day.

### Tissue samples

Canine mammary tumor tissue sections were derived from the archives of the Department of Pathology and Veterinary Diagnostics, Faculty of Veterinary Medicine, Warsaw University of Life Sciences –WULS (Poland). The samples were surgically obtained during the mastectomy from 50 female dogs of various breeds. The tissue samples were fixed in 8% neutral buffered formalin and routinely embedded in paraffin. Eighteen canine mammary tumors were snap frozen using liquid nitrogen and archived at -80°C.

Most of the cases’ information about the presence/absence of metastases was known. The tumors that gave metastases were surgically removed together with the metastatic site. The presence of neoplastic cells on the metastatic site was histologically confirmed. The immunohistochemical examination of cytokeratin, vimentin, smooth muscle actin, s100 protein and p63 protein expression was performed (data not shown). Histological diagnoses were achieved on haematoxylin and eosin (HE) stained slides according to the World Health Organization (WHO) Histological Classification and Mammary Tumors of the Dog and Cat classification [26]. Tumor grading was based on the Misdorp [27] classification and was evaluated in respect to tubule formation, degree of differentiation and mitotic index as: the 1^st^, the 2^nd^ and the 3^rd^ grade of malignancy. The tumors were classified as benign adenomas or malignant carcinomas (simple carcinomas or complex carcinoma). The tumors were divided into five categories: benign adenomas (n = 10), malignant adenocarcinomas: of the 1^st^ grade of malignancy (n = 10), of the 2^nd^ grade of malignancy (n = 10), of the 3^rd^ grade of malignancy (n = 10) and metastatic (n = 10) (which gave local or distant metastases).

### Immunohistochemistry (IHC)

Four micrometer (4 μm) sections from paraffin blocks containing tumor tissue were baked in 37°C overnight. After dewaxing in xylene and rehydration in ethanol, for antigen retrieval, the slides were placed in 0.02 M citrate buffer, pH 6.0 and boiled in the decloaking chamber.

The cell lines were cultured on Lab-Tek (Nunc Inc. USA) 4-chamber culture slides for 24 hrs and subjected to the immunohistochemical analysis after ethanol (70%) fixation (10 min).

The samples were incubated in the Peroxidase Blocking Reagent (Dako, Denmark) for 10 min at room temperature prior to the antibody incubation. After 30 min incubation in 5% bovine serum albumin (Sigma Aldrich, Germany), the following primary antibodies were used (diluted in 1% bovine serum): rabbit polyclonal anti-ghrelin (Abcam, United Kingdom) diluted 1:50 and incubated one hour at room temperature; rabbit polyclonal anti-Growth Hormone Secretagogue Receptor (Novus Biologicals, USA) diluted 3 μg/ml and incubated overnight at +4°C. For the staining the EnVision kit (Labelled Polymers consist of secondary anti-rabbit antibodies conjugated with the HRP enzyme complex obtained from Dako) was used. To develop a colored product, the 3,3^′^-Diaminobenzidine (DAB) substrate was used (Dako). Finally, the hematoxyline was used for nuclei counterstaining.

For each immunohistochemical experiment of paraffin slides, positive controls were set aside: canine stomach (for ghrelin) and canine pituitary (for GHS-R). For each experiment of paraffin slides and cell lines the negative control was stained without use of primary antibodies.

Three consecutive tissue sections and four slides of each cell line were analyzed. Ten to 20 pictures of each slide were taken (depending on the sample size) using Olympus microscopy BX60. Only the regions of cancer cells and stroma were analyzed in the primary tumors whereas the necrotic regions were avoided. The number of ghrelin positive cells (brown color) and the colorimetric intensity of the GHS-R expression reflected as IHC-stained antigen spots (brown color) were counted by a computer-assisted image analyzer (Olympus Microimage™ Image Analysis, software version 4.0 for Windows, USA). The antigen spot color intensity is expressed as a mean pixel integrated optical density (IOD) on a 1-40,000 scale.

### Real-time qPCR

Total RNA from the frozen canine mammary tissues and cell lines suspensions was isolated using a Total RNA kit (A&A Biotechnology, Poland) according to the manufacturer’s protocol. Isolated RNA samples were dissolved in RNase-free water. The quantity of isolated RNA was measured using NanoDrop (NanoDrop Technologies, USA). The samples with adequate amounts of RNA were treated with DNaseI to eliminate DNA contamination. The samples were subsequently purified using RNeasy MiniElute Cleanup Kit (Qiagen). Finally RNA samples were analyzed on a BioAnalyzer (Agilent, California, USA) to measure final RNA quality and integrity.

The *ghsr* and *ghrl* primers were designed using PRIMER3 software (free on-line access) and checked using Oligo Calculator (free on-line access) and Primer-Blast (NCBI database). The used sequences for *ghsr* were as follow: 1) TTCCACGTGGGCGATATTTATT and TGGCAGCACTGAGGTAGAAGAGG (to assess *ghsr* expression at mRNA level in examined cell lines and tissues) and CCTGGTATCCTTTGTCCTCTTCT and CTTTCATCCTTCAGAGTGGAGAG (to assess *ghsr* knockdown effect after siRNA treatment). The optimal annealing time for the first pair was 5 sec, whereas optimal annealing temperature was 59°C. The optimal annealing time for the second pair was 10 sec, whereas optimal annealing temperature was 63°C. The used sequences for *ghrl* were as follow: TCAGGTGAGTGCTTCTAGGG and CACTCGGGAAGTTTCTTCAA. The optimal annealing time was 5 sec, whereas optimal temperature was 61°C. *rps19* and *hprt* genes were used as a non-regulated reference for the normalization of target gene expression (sequences and reaction conditions has been previously given an account of: [23-25,28,29].

Quantitative RT-PCR was performed using fluorogenic SYBR Green and the Sequence Detection System, Fast 7500 (Applied Biosystems). Data analysis was carried out using the 7500 Fast System SDS Software Version 1.4.0.25 (Applied Biosystems, USA). The results were analyzed using comparative Ct method [30]. Relative transcript abundance of the gene equals ΔCt values (ΔCt = Ct^reference^ – Ct^target^). Relative changes in transcript are expressed as ΔΔCt values (ΔΔCt = ΔCt^control^ – ΔCt^examined^). The experiment was conducted three times.

### Cell viability assay (MTT-assay)

Cell viability (metabolic activity of viable cells) was quantified by MTT assay. Cells were seeded into 96-well plate (Nunc Inc., Denmark) at the concentration of 6 × 10^4^ cells per well. When cells reached 60-70% confluence, the medium was replaced with medium containing 10% FBS and *n*-octanoylated ghrelin peptide (Peptides International) at the concentrations of 1, 10, 100 and 1000 nM. To protect ghrelin from inactivation, the culture medium contained also 20 nM serine protease inhibitor, 4-(2-aminoethyl) benzenesulphonyl fluoride hydrochloride (AEBSF, Sigma Aldrich) [31]. The cultures were incubated for 24 hours.

In order to determine the mechanism by which ghrelin increased proliferation/viability of the cells (to assess if it acts via GHS-R), they were additionally pre-treated for 1 hr with the 100 and 200 μM of GHS-R-specific antagonist [D-Lys^3^]-GHRP6 (Peptides International). Then, cells were incubated in 0.5 mg/ml tetrazolium salt MTT diluted in phenol red-free RPMI 1640 medium (Sigma Aldrich) for 4 hrs at 37°C. To complete solubilization of the formazan crystals, 100 μl of DMSO (Dimethyl sulfoxide, Sigma Aldrich) was added to each well. Cells viability was quantified by measuring photometric absorbance at 570 nm in multi well plate reader Infinite 200 PRO Tecan™ (TECAN, Mannedorf, Switzerland). All the samples were examined in triplicate, each experiment was conducted five times (n = 15).

### Cell proliferation assay (BrdU incorporation assay)

BrdU cell proliferation assay was carried out according to the BrdU labeling protocol (BD Bioscience, USA). Briefly, canine mammary cancer cells where cultivated in culture flask to reach 60-70% confluence. The medium was then removed and replaced medium containing 10% FBS with 20 nM AEBSF and: (1) ghrelin at the concentration of 1, 10, 100 and 1000 nM or (2) GHS-R-specific peptide antagonist [D-Lys^3^]-GHRP6 (10, 100, 200, 500 and 1000 μM) for 24 hrs. Then, 10 μM of BrdU (BD Bioscience) was added to culture medium for one hour. The cells were washed twice with PBS, harvested by trypsinization, suspended in PBS and fixed in ice-cold ethanol (70%). The cells were then stored at -20°C for 12 hrs and then washed with Wash Buffer (BD Bioscience, USA) and suspended in denaturing solution (2M HCl) for 20 min at room temperature (RT) to denature DNA. The cells were then washed again with Wash Buffer and resuspended in 0.1 M sodium borate (Na_2_B_4_O_7_, Sigma Aldrich, USA) of pH 8.5, for 2 minutes at RT, to neutralize any residual acid. The cells were then washed with Wash Buffer and incubated with FITC-conjugated anti-BrdU antibodies (20 μl) and isotype control (BD Bioscience, USA) for 30 min at RT. Cells were then incubated with 10 μl/ml of propidium iodide (PI) (Sigma Aldrich) for 30 min at RT. All the samples were analyzed using flow cytometry (FACS Aria II, Becton Dickinson, USA). The experiment was conducted in three replicates.

### siRNA transfection

The siRNA transfection procedure on canine mammary cancer cells has been given an account of in our previous paper [25]. However, the procedure was optimized for CMT-W1M cell line. Thus the cell density, transfection reagent toxicity and transfection efficacy has been assessed (according to the procedure described in our previous manuscript: [25]). The canine (*Canis lupus familiaris*) *ghsr* sequence was obtained from Gene Bank with accession number [EF536345.1]. The siRNA duplexes were designed by http://www.sigmaaldrich.com/life-science/custom-oligos/sirna-oligos/sirna-design-service.html. The results were confirmed using two independent algorithms: Dharmacon (OligoWalk) and Ambion and at last two duplexes were chosen for further experiments (obtained from Sigma Aldrich) (1^st^ duplex sequences, ghr1, are as follow: CCAUCAAUCCCAUUCUGUAdTdT and UACAGAAUGGGAUUGAUGGdTdT; 2^nd^ duplex sequences, ghr2, are as follow: GCUCUCCACUCUGAAGGAUdTdT and AUCCUUCAGAGUGGAGAGCdTdT). Each duplex was used at 2 different concentrations (ghr1 or ghr2, and ghr1^′^ or ghr2^′^ at concentration of 6 or 12 pmol, respectively) the mixture of both duplexes was also used (ghr1 + ghr2: 6 pmol + 6 pmol). The RNA interference has been assessed by Real-time qPCR. Transfected and control CMT-W1M cells (cells cultured according to the transfection procedure with transfection reagent + non-coding RNA (Negative Universal Control, Invitrogen)) were scraped, and the total RNA from the cell suspension samples was isolated. Because during optimization of the siRNA transfection procedure for CMT-W1M cell line any significant changes in cells viability between control cells and cells treated with only transfection reagent has been observed (data not shown), the mock-transfected control has been omitted.

### Apoptosis assay

The Annexin V-FITC and propidium iodide (PI) dual staining was applied for apoptosis analysis. Normal cells and cells treated with (1) ghrelin at the concentration of 1, 10, 100 and 1000 nM and 20 nM AEBSF, (2) inhibitor of ghrelin receptor [D-Lys^3^]-GHRP6 at the concentration of 10 μM, 100 μM, 200 μM, 500 μM and 1000 μM for 24 hrs (3) transfection reagent + non-coding RNA (as a control for transfected cells) and (4) *ghsr* siRNA, were harvested by trypsinization and together with the cells floated in medium were stained using an Annexin V Kit (Becton Dickinson, USA), according to the manufacturer’s protocol. The cells were immediately (within 1 hr) analyzed by flow cytometry (BD FACS Aria II, Becton Dickinson, USA). Early apoptotic cells with exposed phosphatidylserine but intact cell membranes bound to Annexin V-FITC but excluded PI. Cells in late apoptotic stages were labeled with both Annexin V-FITC and PI, whereas necrotic cells were labeled with PI only. All samples were assayed in triplicate, and each experiment was performed three times (n = 9).

### *In vitro* wound healing assay (scratching test)

Scratch assay was conducted to assess the influence of ghrelin on canine mammary carcinoma cell motility. The cells were seeded in a high density at 600 mm Petri dishes (Corning-Costar, Cambridge, MA, USA). After 24 hrs, the medium was removed and replaced medium containing 10% FBS with 20 nM AEBSF and (1) ghrelin at concentration of 1, 10, 100 and 1000 nM or (2) specific GHS-R inhibitor [D-Lys^3^-GHRP6 at concentration of 10 uM, 100 μM, 200 uM, 500 μM and 1000 μM for 24 hrs. When the cells reached full confluence, the monolayers were wounded by scratching the surface as uniformly and straight as possible with a pipette tip (1000 μl) at least three times. The images of cells invading the scratch were captured at indicated time points (0, 3, 6, 9, 12 and 24 hrs) using phase contrast microscopy (IX 70 Olympus Optical Co., Germany). The pictures have been analyzed using a computer-assisted image analyzer (Olympus Microimage™ Image Analysis, software version 4.0 for Windows, USA). The migration rate was expressed as percentage of scratch closure and was calculated as follows: % of scratch closure = a-b/a, where (a) is a distance between edges of the wound, and (b) is the distance which remained cell-free during cell migration to close the wound [32]. The values are the means of three independent wound fields from three independent experiments (n = 9).

### Invasion and migration assay

The BD BioCoat™ 24-Multiwell Invasion System (BD Biosciences, USA) pre-coated with BD Matrigel™ Matrix were used according to the manufacturer’s protocol. The insert plates were prepared by rehydrating the BD Matrigel™ Matrix layer with phosphate buffered saline (PBS) for two hours at 37°C. The rehydration solution was then carefully removed, and 500 μl of cell suspension was added to the apical chambers (2.5 × 10^5^ cells). Cell suspension was prepared by trypsinizing cell monolayers (80% confluent) and resuspending the cells in RPMI 1640 medium containing 0.1% FBS, 20 nM of AEBSF and ghrelin at the concentration of 1, 10 and 100 nM. Then 750 μl of chemoattractant (10% FBS) was added to the each of the basal chambers. Assay plates were incubated for 22 hours at standard culturing conditions. Followed incubation medium was carefully removed from apical chamber and insert system was transferred into a second 24-well plate containing 500 μl of 2.5 μg/ml Calcein AM in Hanks’ Balanced Salt solution (HBSS). Plates were incubated one hour at standard culturing conditions. Then the fluorescence of invaded cells was measured at excitation wavelength 485 nm and emission wavelength 530 nm using florescent plate reader with bottom reading capabilities Infinite 200 PRO Tecan™ (TECAN, Switzerland). All samples were assayed in triplicate, and each experiment was conducted three times (n = 9).

To evaluate migratory potential the BD Falcon™ FluoroBlock™ 24-Multiwell Insert Plates (8 micron pore size) (BD Biosciences, USA) was used. The determination protocol for the canine mammary cancer cells migration was the same as the invasion assay, with the exception that no Matrigel was used and rehydrating of the plate was omitted. In order to determine fluorescence of cells invaded through membrane coated by Matrigel fluorescence microscopic analysis using Olympus microscopy BX60 at x4 magnifications was applied.

### Statistical analysis

The statistical analysis was conducted using Prism version 5.00 software (GraphPad Software, California, USA). The one-way ANOVA and Tukey HSD (Honestly Significant Difference) post-hoc test, Dunnett’s test and *t*-test were applied as well as regression analysis. The p-value < 0.05 was regarded as significant whereas p-value < 0.01 and p-value < 0.001 as highly significant. The data was expressed as means +/- S.E.M. unless otherwise stated. The *in vitro* wound healing assay was analyzed using two-way RM ANOVA and Bonferroni post-hoc test.

## Results

### Expression of ghrelin and GHS-R in canine mammary tumors and in canine mammary carcinoma cell lines

**Figure 1:**
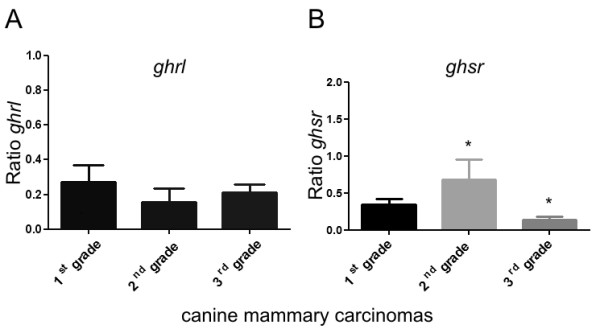
**Ghrelin and growth hormone secretagogue receptor expression at mRNA level in canine mammary adenocarcinomas.** The relative gene expression level (ratio) of (**A**) ghrelin and (**B**) growth hormone secretagogue receptor in adenocarcinomas of the 1^st^, the 2^nd^, the 3^rd^ grade of malignancy (total n = 18) determined using Real-time PCR. The result are presented as a mean (±SEM) from 6 tumors in each group. The statistical analysis was performed using Prism version 5.00 software (GraphPad Software, USA). The one-way ANOVA and Tukey HSD post-hoc test were applied. p < 0.05 was regarded as significant and marked as *.

The expression of ghrelin and growth hormone secretagogue receptor at mRNA level was detected in adenocarcinomas of the 1^st^, the 2^nd^ and the 3^rd^ grade of malignancy (total n = 18). No significant differences in ghrelin mRNA level have been observed between these groups (Figure 1A), however mRNA level of growth hormone secretagogue receptor was significantly lower (p < 0.05) in tumors of the 3^rd^ grade of malignancy compared to the tumors of the 2^nd^ grade of malignancy (Figure 1B).

**Figure 2:**
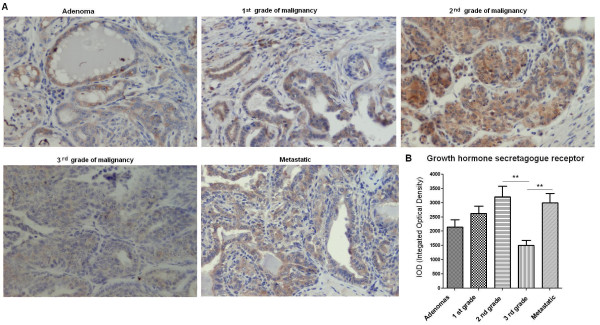
**Ghrelin expression in canine mammary tumors.** (**A**) Light micrographs of canine mammary adenomas, adenocarcinomas of the 1^st^, the 2^nd^, the 3^rd^ grade of malignancy and tumors that gave local/distal metastases (total n = 50) obtained with Olympus BX60 microscope (at the magnification of x20). The ghrelin antigen is represented by brown colored precipitate in cytoplasm. (**B**) The graph of integrated optical density (IOD) of ghrelin-positive cells in canine mammary tumors. The colorimetric intensity of the IHC-stained antigen spots was evaluated by a computer-assisted image analyzer (Olympus Microimage™ Image Analysis, software version 5.0 for windows, USA). Ten to 20 pictures in each slide were analyzed. The result are presented as a mean (±SEM) from 10 tumors in each group. The statistical analysis was performed using Prism version 5.00 software (GraphPad Software, USA). The one-way ANOVA and Tukey HSD post-hoc were applied to analyze the results. p < 0.05 was regarded as significant and marked as *.

The expression of ghrelin peptide was detected in both benign and malignant canine mammary tumors. The analysis showed that ghrelin immunoreactivity was significantly lower (p < 0.05) in benign tumors (adenomas) comparing to the tumors that gave local or distant metastases (carcinomas) (1571 ± 183.5 and 2612 ± 345.9, respectively) (Figure 2A, B). The number of ghrelin-positive cells increased in the group of non-metastatic tumors from benign to the most malignant samples, however no significant differences between these groups have been observed (Figure 2A, B).

**Figure 3:**
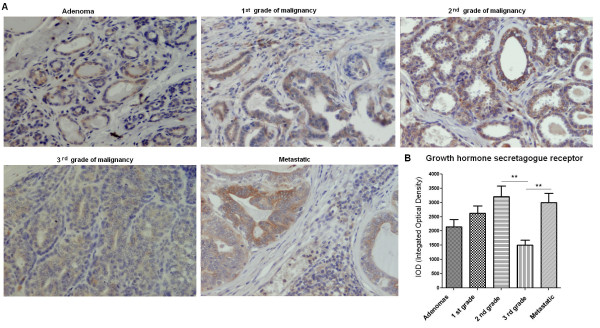
**Growth hormone secretagogue receptor expression in canine mammary tumors.** (**A**) Light micrographs of canine mammary adenomas, adenocarcinomas of the 1^st^, the 2^nd^, the 3^rd^ grade of malignancy and tumors that gave local/distal metastases (total n = 50) obtained with Olympus BX60 microscope (at the magnification of x20). The growth hormone secretagogue receptor (GHS-R) antigen is represented by brown colored precipitate in cytoplasm. (**B**) The graph of integrated optical density (IOD) of growth hormone secretagogue receptor (GHS-R)-positive cells in canine mammary tumors. The colorimetric intensity of the IHC-stained antigen spots was evaluated by a computer-assisted image analyzer (Olympus Microimage™ Image Analysis, software version 5.0 for windows, USA). Ten to 20 pictures in each slide were analyzed. The result are presented as a mean (±SEM) from 10 tumors in each group. The statistical analysis was performed using Prism version 5.00 software (GraphPad Software, California, USA). The one-way ANOVA and Tukey HSD post-hoc were applied to analyze the results. p- < 0.01 was regarded as highly significant and marked as **.

Immunohistochemistry also demonstrated expression of growth hormone secretagogue receptor (GHS-R) in all benign and malignant canine mammary tumors. Significantly lower expression (p < 0.01) of this antigen was found in tumors of the 3^rd^ grade of malignancy (1500 ± 182.6) than in tumors of the 2^nd^ grade of malignancy or in metastatic tumors (3204 ± 377.6 and 2990 ± 335.9, respectively) (Figure 3A, B). No significant differences have been observed between the other tumor groups.

**Figure 4:**
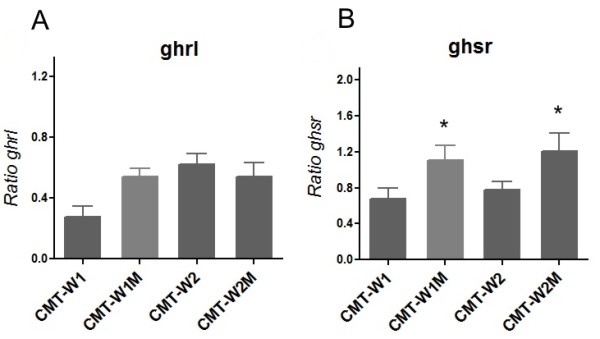
**Ghrelin and growth hormone secretagogue receptor expression at mRNA level in canine mammary carcinoma cell lines.** The relative gene expression level (ratio) of (**A**) ghrelin and (**B**) growth hormone secretagogue receptor in canine mammary carcinoma cell lines (CMT-W1, CMT-W2) and their lung metastases (CMT-W1M and CMT-W2M) determined using Real-time qPCR. The result are presented as a mean (±SEM). The statistical analysis was performed using Prism version 5.00 software (GraphPad Software, USA). The one-way ANOVA and Tukey HSD post-hoc were applied to analyze the results. p < 0.05 was regarded as significant and marked as *.

Real-time PCR analysis showed ghrelin and growth hormone secretagogue receptor mRNA expression in all the examined cell lines (Figure 4A, B). Growth hormone secretagogue receptor expression was significantly higher in the cell lines isolated from mammary carcinoma metastases to the lungs (see also our previous paper: [23]).

**Figure 5:**
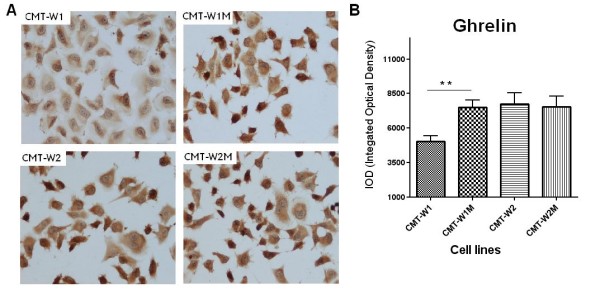
**Ghrelin expression in canine mammary carcinoma cell lines.** (**A**) Representative pictures of canine mammary adenocarcinoma cell lines (CMT-W1, CMT-W2) and their lung metastases (CMT-W1M and CMT-W2M). The majority of the cancer cells showed a strong cytoplasmic staining reaction (brown color) of ghrelin (anti-pre proghrelin antibody obtained from Abcam, UK was used). Pictures were obtained with Olympus BX60 microscope (at the magnification of x20). (**B**) The graph of integrated optical density (IOD) of the ghrelin-positive cells in examined canine cancer cell lines. The colorimetric intensity of the IHC-stained antigen spots was evaluated by a computer-assisted image analyzer (Olympus Microimage™ Image Analysis, software version 5.0 for windows, USA). Twenty pictures in each slide were analyzed. The results are presented as a mean (±SEM) from tree separate experiments. The statistical analysis was performed using Prism version 5.00 software (GraphPad Software,USA). The one-way ANOVA and unpaired *t*-test were applied. p < 0.01 was regarded as significant and marked as **.

**Figure 6:**
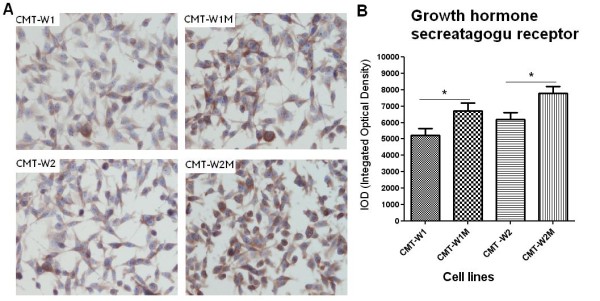
**Growth hormone secretagogue receptor expression in canine mammary carcinoma cell lines.** (**A**) Representative pictures of canine mammary adenocarcinoma cell lines (CMT-W1, CMT-W2) and their lung metastases (CMT-W1M and CMT-W2M). Cancer cells showed a cytoplasmic staining reaction (brown color) of growth hormone secretagogue receptor (anti-Growth Hormone Secretagogue Receptor antibody obtained from Santa Cruz Biotechnology, USA was used). Pictures were obtained with Olympus BX60 microscope (at the magnification of x10). (**B**) The graph of integrated optical density (IOD) of growth hormone secretagogue receptor (GHS-R)-positive cells in examined canine cancer cell lines. The colorimetric intensity of the IHC-stained antygen spots was evaluated by a computer-assisted image analyzer (Olympus Microimage™ Image Analysis, software version 5.0 for windows, USA). Twenty pictures in each slide were analyzed. The results are presented as a mean (±SEM) from tree separate experiments. The statistical analysis was performed using Prism version 5.00 software (GraphPad Software, USA). The one-way ANOVA and unpaired *t*-test were applied. p < 0.05 was regarded as significant and marked as *.

Immunohistochemical examination confirmed expression of both peptides in all of the examined cell lines (Figures 5, 6). Significantly lower expression (p < 0.01) of ghrelin antigen was detected in cell line isolated from primary canine mammary adenocarcinoma (CMT-W1) than in the cell line isolated from its metastasis to the lungs (CMT-W1M) (5030 ± 428.4 and 7494 ± 548.3, respectively). The immunoreactivity of ghrelin was similar in another pair of the cell lines (7764 ± 778.5 and 7537 ± 773.1 in CMT-W2 and CMT-W2M, respectively) (Figure 5A, B). However, GHS-R immunoreactivity was significantly lower (p < 0.05) in both canine mammary carcinomas isolated from primary tumors than in cell lines isolated from their metastases to the lungs: 5230 ± 399.5 and 6729 ± 462.7 in CMT-W1 and CMT-W1M cell lines, respectively and 6189 ± 433.0 and 7792 ± 401.9 in CMT-W2 and CMT-W2M cell lines, respectively (Figure 6A, B).

### Ghrelin enhances viability and proliferation of canine mammary cancer cells

**Figure 7:**
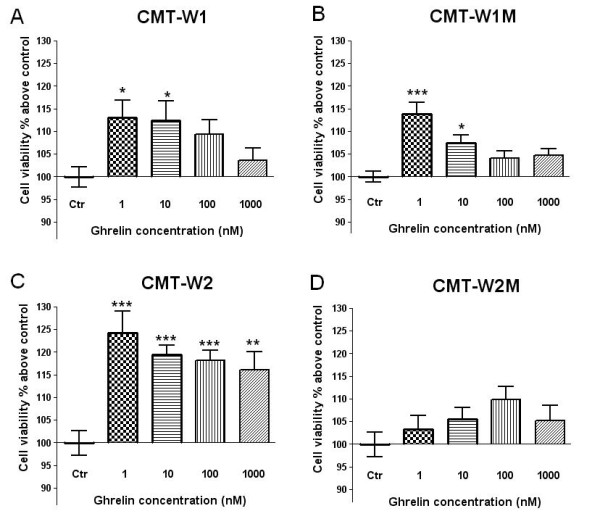
**Ghrelin affects viability of canine mammary carcinoma cells.** The canine mammary cancer cells viability determined by MTT assay in control conditions (ctrl) and in a presence of *n*-octanoylated ghrelin peptide (Peptides International) at concentrations of 1, 10, 100 and 1000 nM for 24 h. Absorbance at 570 nm has been converted to percentages above control (±SEM). The experiment has been conducted in n = 15 repetitions. Ghrelin treatment at the concentration of 1 and 10 nM; 1 and 100 nM, 1-1000 nM significantly increased cell viability of the CMT-W1 (**A**), CMT-W1M (**B**) and CMT-W2 (**C**) cell lines, respectively. No significant differences has been observed in CMT-W2M cell line (**D**). The statistical analysis was performed using Prism version 5.00 software (GraphPad Software, USA). One-way ANOVA followed by Dunnett’s post hoc comparisons were applied. p < 0.05 was marked as *, p < 0.01 was marked as ** and p < 0.001 was marked as ***.

**Figure 8:**
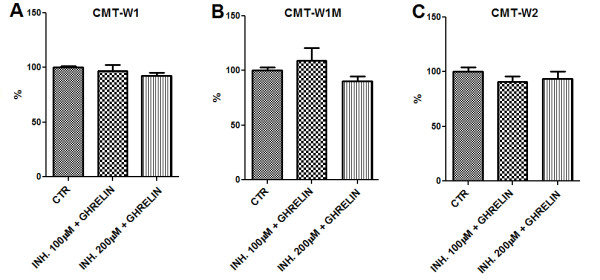
**Canine mammary carcinoma cells response for ghrelin was completely abolished after pre-treatment with specific inhibitor of GHS receptor ([D-Lys**^**3**^**]-GHRP6).** The canine mammary cancer cells viability determined by MTT assay in control conditions (CTR) and in one hour pre-incubation with specific inhibitor of GHS receptor ([D-Lys^3^]-GHRP6) at concentrations of 100 and 200 μM and following incubation with *n*-octanoylated ghrelin peptide (10 nM) (Peptides International) for 24 hrs. Absorbance at 570 nm has been converted to percentages (±SEM). The experiment has been conducted in n = 15 repetitions. The pre-treatment with specific inhibitor of GHS receptor completely abolished the cells response for ghrelin treatment (10 nM) in the CMT-W1 (**A**), CMT-W1M (**B**) and CMT-W2 (**C**) cell lines, respectively. The statistical analysis was performed using Prism version 5.00 software (GraphPad Software, USA). One-way ANOVA followed by Dunnett’s post hoc comparisons were applied. No significant differences has been observed.

Ghrelin treatment increased cells viability (determined by MTT assay) and cells proliferation (assessed using BrdU incorporation assay) in three of the examined cell lines except the CMT-W2M cell line. The viability of the CMT-W1 cells was significantly increased by approximately 14% when treated with 1 nM *n*-octanoylated ghrelin (p < 0.05) and 12% when treated with 10 nM ghrelin (p < 0.05) compared to control cells (Figure 7A). Ghrelin treatment at dose of 1 nM and 10 nM also increased cell viability in the CMT-W1M cell line (14% above control, p < 0.01 and 8% above control, p < 0.05; respectively) (Figure 7B). The highest response for ghrelin treatment was observed in the CMT-W2 cell line. The viability of the CMT-W2 cells was significantly increased when treated with ghrelin at the concentration of 1 nM (p < 0.01), 10 nM (p < 0.01), 100 nM (p < 0.001) and 1000 nM (p < 0.01) by approximately 25%, 19%, 18% and 16%, respectively (Figure 7C). Metabolic activity of the CMT-W2M cells was not significantly changed after incubation with ghrelin (Figure 7D). The pre-treatment of cells for 1 hr with specific inhibitor of GHS receptor ([D-Lys^3^]-GHRP6) completely abolished the cells response for ghrelin (Figure 8) what confirmed that in examined cell lines ghrelin acts via GHS-R.

**Figure 9:**
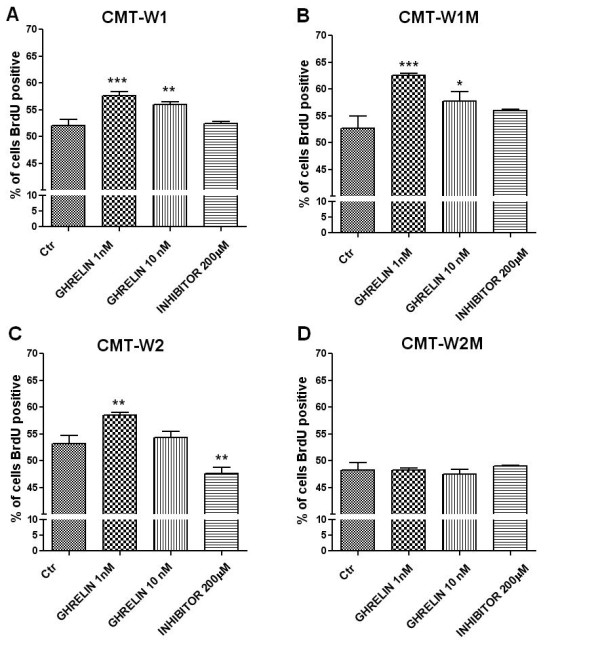
**Ghrelin stimulates proliferation of canine mammary carcinoma cells.** The effect of *n*-octanoylated ghrelin peptide (Peptides International) and GHS-R-specific peptide antagonist [D-Lys^3^]-GHRP6 (Peptides International) treatment on proliferation of canine mammary cancer cells assessed by BrdU incorporation test (BD Bioscience, USA). The number of proliferating cells represented as a percentage of BrdU-positive cells (±SD) obtained with FACS Aria II (Becton Dickinson) is significantly increases after the ghrelin treatment at concentration of 1 and 10nM in CMT-W1 (**A**), and CMT-W1M (**B**) cell line whereas at concentration of 1 nM dose in CMT-W2 (**C**). In this cell line a decrease in number of proliferating cells after ghrelin receptor inhibitor treatment (200μM) has been observed. No significant differences has been observed in CMT-W2M cell line (**D**) nor after the ghrelin treatment, neither after the ghrelin receptor inhibitor treatment. The experiment has been conducted in three repetitions. The statistical analysis was performed using Prism version 5.00 software (GraphPad Software, USA). One-way ANOVA followed by Tukey HSD post-hoc test were applied. p < 0.05 was marked as *, p < 0.01 was marked as ** and p < 0.001 was marked as ***.

The role of ghrelin on cellular proliferation was confirmed by BrdU incorporation assay. Ghrelin treatment at 1 nM and 10 nM doses increased proliferation of cancer cells to 57.6 ± 0.8% and 55.9 ± 0.5% (p < 0.001) in CMT-W1 cell line and to 62.5 ± 0.4% and 57.7 ± 1.8% (p < 0.01) in CMT-W1M cell line (Figure 9A, B). Grelin treatment at the concentration of 1 nM caused increase of proliferation to 58.5 ± 0.6% (p < 0.01) in CMT-W2 cell line, however treatment with specific GHS-R inhibitor at 200 μM dose induced anti-proliferative effect, which was not observed in other examined cell lines (Figure 9C). Cell proliferation was not affected by ghrelin in the CMT-W2M cell line (Figure 9D).

In both experiments it was impossible to create a dose–response curve. The results indicated that smaller doses of ghrelin (1-10 nM) significantly increased cancer cells proliferation, however any dose-dependent effect was not observed (regression analysis).

### *ghsr* RNA interference

Based on the MTT assay results, the optimal cell number for transfection experiment was 100 000 per well on 96-well plate. Culture of 100 000 cells per well at the beginning of the experiment ensured 60% confluence (due to absorbance) at the day of transfection. Transfection reagent toxicity evaluation revealed that in case of CMT-W1 cell line the best viability results were reached using Lipofectamine RNAiMAX (Invitrogen, USA). The highest viability of the cells (the same as in control cells, due to absorbance) was reached with Lipofectamine RNAiMAX at the concentration of 0.8 μl per well.

The confocal microscopy observations and subsequent computer-assisted image analysis (Olympus Microimage™ Image Analysis, software version 4.0 for Windows, USA) revealed that Lipofectamine RNAiMAX at the concentration of 0.8 μl per 100 000 cells per well proved a highly effective transfection reagent ensuring the transfection efficiency at the level of 90%. Consequently, experiments were conducted under the same conditions.

The control cells used for further experiments were cultured under the transfection conditions with tranfection reagent + non-coding RNA.

The real-time qPCR and immunohistochemistry results confirmed the effect of *ghsr* gene silencing. The lowest expression of *ghsr* (45.07% reduction in expression (when compared against control cells) was found in the cells treated with the mixture of ghsr1 and ghsr2 sequences (p < 0.001). Only about 15-28% reduction of *ghsr* expression, comparing against the control cells, was observed in case of ghsr1 sequence given at two concentrations (p < 0.05). We observed 27.51% and 48.69% reduction of expression compared to control cells after the ghsr2 and ghsr2^′^ interference, respectively (p < 0.05 and p < 0.01, respectively).

The immunohistochemical studies revealed higher efficacy of gene silencing at the protein level than at the mRNA level. The most effective was ghsr1 sequence causing 84.41% reduction in protein levels compared against the control cells. Ghrs1^′^ sequence caused 68.72% of protein expression reduction, whereas ghsr2 sequence caused 56.17% reduction. Ghsr2^′^ and mixture of ghsr1 and ghsr2 sequences caused 60.47% and 60.75% reduction of protein expression. All these results have been statistically significant at the level of p < 0.05. For apoptosis analysis the ghsr1 siRNA duplex was used.

### Ghrelin is an anti-apoptotic factor in canine carcinoma cell lines

**Figure 10:**
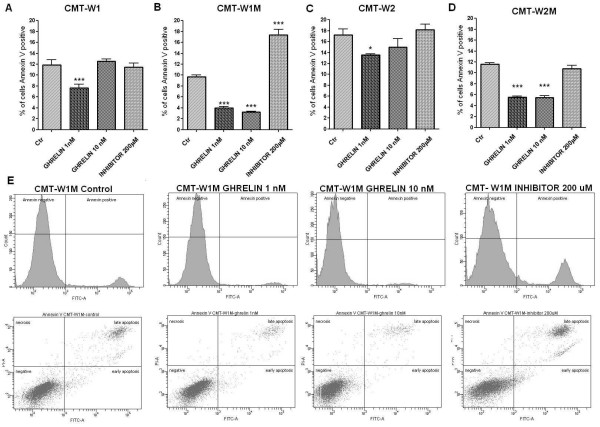
**Ghrelin inhibits basal apoptosis of canine mammary carcinoma cells.** The effect of *n*-octanoylated ghrelin peptide (Peptides International) and GHS-R-specific peptide antagonist [D-Lys^3^]-GHRP6 (Peptides International) treatment on apoptosis of CMT-W1 (**A**), CMT-W1M (**B**), CMT-W2 (**C**) and CMT-W2M (**D**) cell lines, assessed by the Annexin V/PI test (BD Bioscience, USA). The number of apoptotic cells represented as a percentages of Annexin-V-positive cells (±SD) obtained with FACS Aria II (Becton Dickinson) is significantly decreased after the 1nM ghrelin treatment in CMT-W1, and CMT-W2 cell lines and after 1 nM and 10 nM ghrelin treatment in CMT-W1M, and CMT-W2M cell lines. The administration of ghrelin receptor inhibitor ([D-Lys3]-GHRP6) at concentration of 200 μM significantly increased the number of apoptotic cells in the CMT-W1M cell line. The experiment has been conducted in three replicates. p < 0.05 was marked as *, p < 0.01 was marked as ** and p < 0.001 was marked as ***. One-way ANOVA followed by Tukey HSD post-hoc test were applied. (**E**) The representative histograms and cytograms of CMT-W1M cell line double stainined with AnnexinV-FITC and propidium iodide (PI). Cells located on the right side of the histograms represented apoptotic cells. On the cytograms are showed normal, early apoptotic, late apoptotic and necrotic cells. Left bottom quadrant shows normal cells, top left quadrant shows necrotic cells (stained with PI only; damaged cell membrane but no phosphatydilserine exposure), right bottom quadrant shows the early apoptotic cells (stained with Annexin V only; intact cell membrane) and top right quadrant shows cells in late stage of apoptosis (stained with Annexin V and PI - phosphatydilserine exposure and damaged cell membrane).

**Figure 11:**
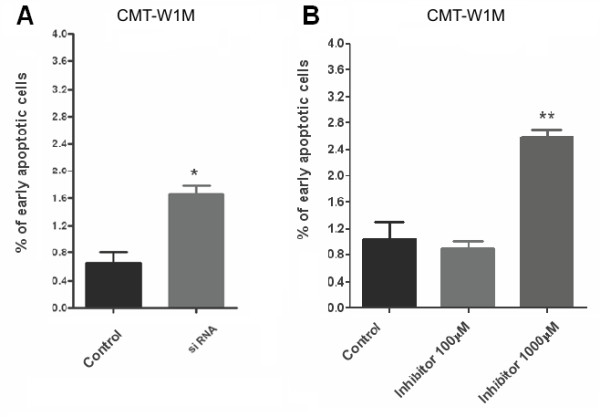
**The impact of siRNA and GHS-R-specific peptide antagonist on early apoptosis of canine mammary carcinoma cells.** (**A**) The effect of knock-down of growth hormone secretagogue receptor by siRNA and (**B**) GHS-R-specific peptide antagonist [D-Lys^3^]-GHRP6 (Peptides International) (1000 μM) on early apoptosis of CMT-W1M cell line assessed by the Annexin V/PI test (BD Bioscience, USA). The number of early apoptotic cells represented as a percentages of Annexin V stained cells (±SD) with intact cell membrane obtained with FACS Aria II (Becton Dickinson) highly significantly increased after a ghrelin inhibitor treatment (p < 0.01 marked as **) and significantly increased after siRNA treatment (p < 0.05 marked as *). The experiment has been conducted in three replicates. The statistical analysis was performed using Prism version 5.00 software (GraphPad Software, USA). One-way ANOVA followed by Tukey HSD post-hoc test and *t*-test were applied.

For analysis of ghrelin influence on apoptosis, the Annexin V and PI double staining was applied. The number of apoptotic cells represented as a percentages of Annexin-V-positive cells was significantly decreased after the ghrelin treatment at the dose of 1 nM (decreased from 11.9 to 7.7%) in the CMT-W1 cell line (p < 0.001) and in the CMT-W2 cell line (p < 0.05) (decreased from 17.2% to 13.5%) (Figure 10A, C). Anti-apoptotic effect of ghrelin was higher in the cell lines isolated from lung metastases of mammary cancer. In control conditions 9.7% and 11.6% of cells has been identified as apoptotic in CMT-W1M and CMT-W2M cell line, respectively. After ghrelin treatment at the concentration of 1 nM and 10 nM the number of apoptotic cells decreased to 4% and 3.2% (respectively) in the CMT-W1M cell line and to 5.6% and 5,4% (respectively) in the CMT-W2M cell line. The statistical analysis showed that differences were highly significant (p < 0.001) (Figure 10B, D). Among all of the examined cell lines only in CMT-W1M cell line incubation with 200 μM GHS-R-specific peptide inhibitor ([D-Lys^3^]-GHRP6) for 24hrs significantly increased the number of apoptotic cells (17.3%, p < 0.001) (Figure 10B, E). In order to investigate if this effect was caused by toxicity of this peptide, we knocked-down the *ghsr* expression by RNAi to confirm the functional importance of the GHS-R in apoptosis in CMT-W1M cell line. Our results showed that siRNA treatment significantly increased number of early apoptotic cells in CMT-W1M cell line (p < 0.05) up to 1.7% (Figure 11A). Cancer cells treatment with GHS-R inhibitor increased number of early apoptotic cells highly significantly (p < 0.01) up to 2.6% (Figure 11B). These differences may be caused by not completely knock-down of GHSR expression (about 15% of cells remained untransfected).

### Ghrelin promotes migration and invasion of canine mammary carcinoma cells

**Figure 12:**
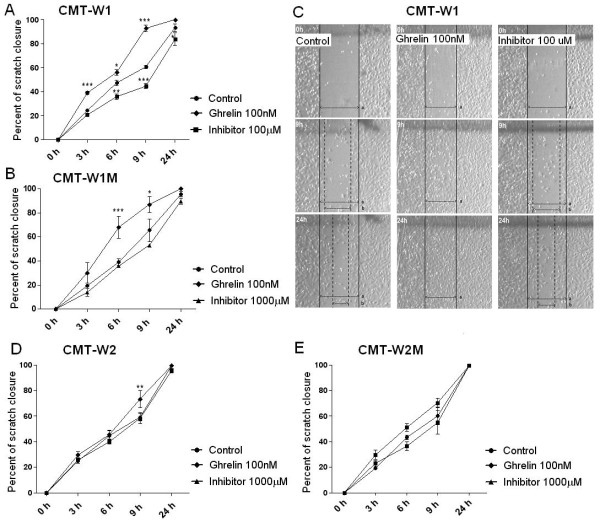
**Wound healing assay of canine mammary carcinoma cells after ghrelin treatment.** Quantification of migration of CMT-W1 (**A**), CMT-W1M (**B**) CMT-W2 (**D**), CMT-W2M (**E**) cell lines represented as percentages of scratch closure and calculated as follows: % of scratch closure = a-b/a, where (a) is a distance between edges of the wound, and (b) is the distance which remained cell-free during cell migration to close the scratch. Ghrelin treatment at the concentration of 100nM increased cells motility after 9 and 24 hrs in the CMT-W1, CMT-W1M and CMT-W2 cell lines. Administration of ghrelin receptor inhibitor (100μM) decreased the motility of the CMT-W1 cells, whereas had no effect on motility of the cells of remaining cell lines. Photographs of cells invading the scratch were taken at the starting point and after 3, 6, 9, and 24 hrs using phase contrast microscopy (IX 70 Olympus Optical Co., Germany). (**C**) Representative pictures of scratch closure in control conditions, after ghrelin treatment (100nM) and GHS-R-specific peptide inhibitor [D-Lys^3^]-GHRP6 (100 μM) treatment in CMT-W1 cell line obtained using Olympus BX60 microscope (x4). Pictures have been analyzed using a computer-assisted image analyzer (Olympus Microimage™ Image Analysis, software version 5.0 for Windows, USA). The experiment has been conducted three times. Two-way ANOVA and Bonferroni post-hoc tests were applied. p < 0.05 was marked as *, p < 0.01 was marked as ** and p < 0.001 was marked as ***.

The wound healing assay showed that ghrelin treatment at the concentration of 100 nM increased migratory abilities of the CMT-W1, CMT-W1M and CMT-W2 cell lines (Figure 12A, B, C, D), however, no effect on CMT-W2M cells has been observed (Figure 12E). CMT-W1 cells incubated with ghrelin (100 nM) almost completely closed the wound (93 ± 2.7%) in 9 hours after scratching, whereas CMT-W1 control cells after 9 hrs closed only 60.9 ± 1.4% of the wound (Figure 12A). Similarly, CMT-W1M cells incubated with ghrelin (100 nM) closed the wound at the level of 86.9 ± 6.6% in 9 hours after scratching, whereas CMT-W1M control cells after 9 hrs closed only 65.6 ± 9.4% of the wound (Figure 12B). Also, CMT-W2 cells incubated with ghrelin (100 nM) closed the wound at the level of 73.6 ± 6.2% in 9 hours after scratching, whereas CMT-W2 control cells after 9 hrs closed only 59.4 ± 2.9% of the wound (Figure 12D). Administration of GHS-R-specific peptide inhibitor ([D-Lys^3^]-GHRP6) at 100 μM dose decreased the motility of the CMT-W1 cells, whereas had no effect on motility of the CMT-W1M or CMT-W2 cells even at the concentration of 1000 μM. CMT-W1 cells incubated with ghrelin receptor inhibitor (100 μM) closed 44,7 ± 2.0% and 83,8 ± 5.3% of the wound in 9 and 24 hrs after scratching (respectively) (Figure 12A, C). CMT-W2M control cells and ghrelin (100 nM) treated cells closed 60,2 ± 6,8% and 70,3 ± 3,6% of the wound (respectively) in 9 hours after scratching, however the difference was not statistically significant (Figure 12E). CMT-W2M completely (100%) closed the wound after 24 hours after scratching in control conditions and after ghrelin treatment. GHS-R-specific peptide inhibitor ([D-Lys^3^]-GHRP6) also had no effect on motility of those cells (Figure 12E).

**Figure 13:**
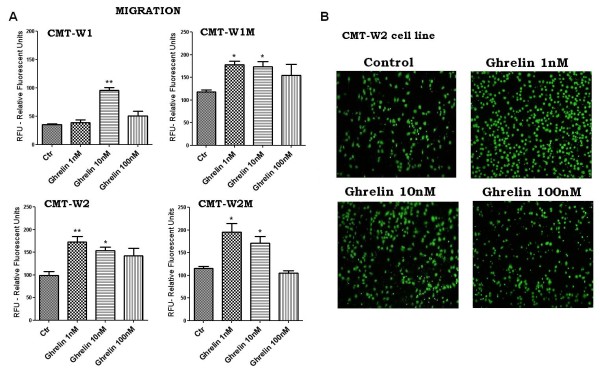
**Ghrelin promotes migration of canine mammary carcinoma cells.** (**A**) Quantification of migration of examined cell lines (CMT-W1, CMT-W1M, CMT-W2, CMT-W2M) represented as relative florescent units obtained by florescent plate reader Infinite 200 PRO Tecan™ (Ex. 485, Em. 530). Ghrelin treatment for 24 hrs at the concentration of 1 nM and 10 nM increased cancer cells migration through membrane of Boyden chamber assay in CMT-W1M, CMT-W2, CMT-W2M cell lines. In CMT-W1 cell line only grelin concentration of 10 nM was effective. The experiment has been conducted three times. The statistical analysis was performed using Prism version 5.00 software (GraphPad Software, USA). One-way ANOVA followed by Tukey HSD post-hoc test and unpaired *t*-test were applied. p < 0.05 was marked as *, p < 0.01 was marked as **. (**B**) Fluorescence microscopic analysis of the migrated CMT-W2 cells using Olympus microscopy BX60 at x4 magnification.

Cell migration assessed in Boyden chambers was increased by incubation with 10 nM of ghrelin in CMT-W1 cell line and in the other cell lines (CMT-W1, CMT-W2, CMT-W2M) by incubation with 1 and 10 nM of ghrelin (Figure 13A).

**Figure 14:**
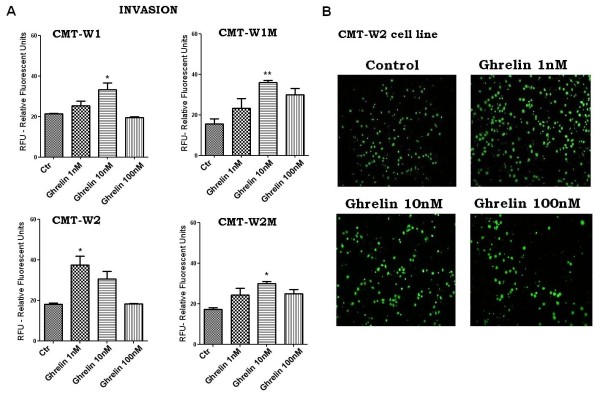
**Ghrelin promotes invasion of canine mammary carcinoma cells.** (**A**) Quantification of invasion of examined cell lines (CMT-W1, CMT-W1M, CMT-W2, CMT-W2M) represented as relative florescent units obtained by florescent plate reader Infinite 200 PRO Tecan™ (Ex. 485, Em.530). Ghrelin treatment for 24 hrs at the concentration of 1 nM in CMT-W2 cell line and of 10 nM in remaining cell lines increased cancer cells invasion through membrane of Boyden chamber system pre-coated by BD Matrigel™ Matrix. The experiment has been conducted three times. The statistical analysis was performed using Prism version 5.00 software (GraphPad Software, USA). One-way ANOVA followed by Tukey HSD post-hoc test and unpaired *t*-test were applied. p < 0.05 was marked as *, p < 0.01 was marked as **. (**B**) Fluorescence microscopic analysis of the invaded through Matrigel layer CMTW-2 cancer cells using Olympus microscopy BX60 at x4 magnification.

Cancer cell invasion was increased by ghrelin treatment for 24 hours. Ghrelin was the most effective at the 10 nM concentration in CMT-W1, CMT-W1M, CMT-W2M, whereas it was the most effective in CMT-W2 cell line at 1 nM concentration (Figure 14A). The analysis conducted using Olympus microscopy BX60 confirmed the results (Figures 13 and 14B).

## Discussion

The co-expression of ghrelin and GHS-R has previously been observed in various neoplasms and related cancer cell lines in humans (for review see: [10]). It may suggest their role in neoplastic cell growth and development through autocrine/paracrine mechanism. However, as far as we realize, there are no published reports regarding similar data on tumors in domestic animals. This is the first report of ghrelin and its receptor expression in canine mammary tumor tissues and cell lines isolated from the canine primary carcinomas (CMT-W1 and CMT-W2) and from their lung metastases (CMT-W1M and CMT-W2M).

We have demonstrated the expression of ghrelin in benign and malignant canine mammary tumors (Figures 1, 2, 3) and in all of the examined cancer cell lines (Figures 4, 5, 6). Interestingly, we observed significantly higher expression of ghrelin in the distant metastases than in the primary carcinomas, which suggest that ghrelin might be involved in mechanism of metastasis (Figure 2). A recent study on human colorectal cancers indicated that ghrelin might initiate malignant transformation and might contribute to the cancer spread [33]. It was also hypothesized that ghrelin mediates the physiological responses of tissues which surround the tumor, to malignant changes [34].

The presence of GHS-R mRNA has been previously detected in all of the examined canine cancer cell lines [23]. Similarly to mRNA expression patterns, GHS-R protein showed significantly higher expression in cell lines isolated from lung metastases of mammary cancer (CMT-W1M and CMT-W2M) what may indicate its role in metastasis (Figures 4, 6). Moreover, we have demonstrated that expression of ghrelin receptor varies in canine mammary tumors depending on the histological grade of malignancy (Figure 3). The GHS-R expression level was higher in well or moderately differentiated cancers of the 1^st^ and the 2^nd^ grade of malignancy compared to poorly-differentiated cancers of the 3^rd^ grade of malignancy. This intriguing finding overlaps with those reported previously, that expression and functionality of specific binding sites for GHS (*growth hormone secreatgogues*) in various types of breast and thyroid carcinomas was maintained in better-differentiated tumors and was decreased in less-differentiated neoplasms [11,12]. Similar expression patterns of GHS-R were observed in human gastric and colorectal cancers [33,34], where the GHS-R expression level was lowest in undifferentiated tumors and correlated inversely with histological grade of malignancy and TNM stage. Moreover, patients with more weight loss showed lower expression of GHS receptor [34]. Therefore, decreased ghrelin receptor expression seemed to correlate with poor prognostic factors such as poor differentiation, advanced stage and malnutrition.

The next step of this study was to assess a role of ghrelin in cancer cell biology. Our results revealed that ghrelin stimulates canine mammary cancer cells proliferation and inhibits apoptosis, possibly via an autocine/paracrine mechanism. Although the role of ghrelin in cancer cells was investigated in multiple experiments using human and rat cancer cell lines, the results were equivocal. Some investigators have found ghrelin as antiproliferative factor [11-14,19], but other have found it a growth stimulator of cancer cells [15-18,20-22]. Cassoni and co-workers described an inhibition of cell proliferation in human thyroid, breast and lung cancer by ghrelin [11,12,14]. Authors found that ghrelin given for 24 hrs at relatively high doses (100 or 1000 nM) decreased cell proliferation. However, after incubation of these cells with ghrelin for 96 hrs, the effect was abolished [11]. Volante et al. [19] have confirmed antiproliferative effect of ghrelin in thyroid cancer cells showing significant growth reduction after 48 hrs and 96 hrs of incubation with ghrelin at the concentration of 100-1000 nM. Interestingly, Cassoni et al. [13] have showed dose-dependent effect of ghrelin treatment in prostate cancer cells. Contrary to the mentioned reports the pro-proliferative effect of ghrelin in several human cancer cell lines has also been well documented. Ghrelin at doses ranging from 1 to 10 nM, which encompasses normal circulating ghrelin levels increased cellular proliferation after 72 h of incubation in breast and prostate cancers [15,16,21]. Ghrelin treatment significantly stimulated cells growth in the highly metastatic (MDA-MB-435) breast cancer cell line up to 36% above control. The most significant proliferative response to ghrelin treatment occurred at the concentrations of 10 and 100 nM in the MDA-MB-231 cell line, and 1–10 nM in the MDA-MB-435 cell line [16]. Thus, these results show that ghrelin given in high doses inhibits cancer cell proliferation, whereas given at low doses increases proliferation. Our results also indicated that ghrelin given at the dose of 1 or 10 nM increased canine mammary cancer cells proliferation in three of four examined cell lines (Figure 7, Figure 9), whereas higher doses caused not significant effect also in three of four examined cell lines. In one cell line, ghrelin did not cause any affect.

To clarify the role of ghrelin in cell proliferation the cell cycle analysis was conducted [22]. This study revealed that although ghrelin did not change the proportion of cells in the G0/G1, S or G2/M phases of cell cycle, it caused a decrease in the number of apoptotic cells in sub-G0 phase. In fact ghrelin was reported to be the antiapoptotic factor in colonic cancer cells [35] as well as in many other types of cells like adipocytes [36], osteoblastic cells [37], or human endothelial cells [38].

The antiapoptotic activity of ghrelin is also supported by our findings (Figure 10). We have showed that ghrelin decreases the number of apoptotic cells in all of the examined canine mammary cancer cell lines (in all of the examined cell lines ghrelin treatment at a dose of 1 nM significantly decreased apoptosis, whereas in two cell lines incubation of cells with the ghrelin concentration of 10 nM also decreased the number of apoptotic cells). Because in CMT-W1M cell line we observed a significant increase in number of apoptotic cells after the treatment with ghrelin inhibitor, our next step was to knock-down the *ghsr* gene to check if the toxic effect was caused by the inhibitor itself or by blocking the GHS-R. Our results showed that treatment of CMT-W1M cell line with *ghsr*-specific siRNA also increased the number of apoptotic cells (early apoptotic) (Figure 11). An increase in number of apoptotic cells was slight, however significant, which can be explained by efficacy of gene silencing. In our experiment only 85% of cells remained transfected.

Recently, a few studies have focused on the involvement of ghrelin in cancer cells motility and ability to metastasis. Duxbury and co-workers [18] investigated the role of ghrelin in metastatic potential of poorly differentiated human pancreatic cancer cells. They found that ghrelin at 10 nM concentration increased not only the proliferation rate of cancer cells but also cellular motility and invasiveness (even up to 60%). Another study showed the promotion of proliferation and invasiveness of human colorectal cancer cells by ghrelin in an autocrine and paracrine manner. This effect was almost completely abolished when the cells were pre-treated with either GHS-R antagonist (D-[Lys3]GHRP6) or a neutralizing ghrelin – specific antibody [33]. Furthermore Dixit et al. [39] revealed that ghrelin acting via functional GHS-R1a caused increase in intracellular calcium mobilization and led to actin polymerization and membrane ruffling, which resulted in an increase of migration and invasion of carcinoma cells.

Our study also showed a role of ghrelin in cancer cells migration and invasion. We have demonstrated that ghrelin given at the concentration of 100 nM increased the migration ability of canine carcinoma cell lines CMT-W1 and CMT-W1M, evaluated by wound healing test (Figure 12). Moreover ghrelin given at the concentration of 1 and/or 10 nM significantly increased migration and invasion of all the examined canine carcinoma cell lines (Figure 13, Figure 14).

The fact that the examined carcinoma cell lines respond in different manner to the various ghrelin doses may be related with differences in endogenous ghrelin production levels. Differences between effective ghrelin doses that stimulate cells proliferation or survival and doses that increase cells migration can be explained by the fact that these different cellular activities are mediated by different cellular pathways. Literature data show that ghrelin increases cellular migration acting on CaMKII, AMPK, and NF-kB pathways [40], whereas increases cellular proliferation and survival acting on ERK1/2 [20]. Thus, probably various ghrelin doses are required to activate various cellular pathways.

## Conclusion

We have showed that ghrelin and GHS-R are differentially expressed in canine mammary tumors. The lowest expression of GHS-R in tumors of the 3^rd^ grade of malignancy may by a potential prognostic factor. Taken together, the data demonstrated that exposure to low doses of ghrelin stimulate cellular proliferation, inhibit apoptosis and promote motility and invasion of canine mammary carcinoma cells. However, further studies are required to investigate the molecular mechanism responsible for ghrelin action in cancer cells. Our study indicates that inhibition of ghrelin/GHS-R axis may be a potential therapeutic target of mammary cancers in dogs in the future.

## Competing interest

The authors declare that they have no competing interests.

## Authors’ contributions

KM: MTT assays, BrdU assay, migration and invasion assays, wound healing assay, immunohistochemistry, statistical analysis, manuscript and figures preparation; KMP: siRNA transfection, Real-time qPCR; EJO: siRNA transfection, Real-time qPCR; ID: pathological examination of mammary carcinomas; JM: RNA isolation, immunohistochemistry; TM: research design; MK: research design, experiments design, apoptosis tests, FACS analyses, manuscript preparation, figures preparation. All authors read and approved the final manuscript.
